# Supplementary material for: Content-rich biological network constructed by mining PubMed abstracts
Source: BMC Bioinformatics. 2004 Oct 8;5:147. doi: 10.1186/1471-2105-5-147 (PMC528731; doi:10.1186/1471-2105-5-147)
Supplement: Additional File 2 — The original results of the above study (non-essential files are deleted to keep the file size under the limit set by BMC bioinformatics). [file 1471-2105-5-147-S2.bz2 › chilibotAdditionalFile2/dip05/354ID1495999E4996/html/IL5_IL5RA.html]

 


 **IL5** and **IL5RA** 
  
Found 67 abstracts in PubMed, retrieved 05.  
 

 What does Google say? 
 PDF only 
| .edu only 

---

**Interactive relationship** (e.g. stimulation, inhibition, etc)

**Neutral relationship**- Using this surface, we quantitated for the first time direct binding of s beta c to IL5R  [ **IL5RA** ]  alpha complexed to either wild type or single chain  **IL5** .  Ref: 12202242 Anal Biochem, 2002
- Biosensor analysis of dynamics of interleukin 5  [ **IL5** ]  receptor subunit beta c interaction with  **IL5**  IL5R  [ **IL5RA** ]  alpha complexes.  Ref: 12202242 Anal Biochem, 2002
- We therefore suggest that an IL 5  [ **IL5** ]  driven inflammation generates an eosinophil tissue phenotype that is characterized by a low IL 5R alpha  [ **IL5RA** ]  expression.  Ref: 12519389 Clin Exp Immunol, 2003
- The IL 5R alpha  [ **IL5RA** ]  was down regulated, in a dose dependent manner, by recombinant IL 5  [ **IL5** ]  and GM CSF, with IL 5  [ **IL5** ]  being most potent.  Ref: 12519389 Clin Exp Immunol, 2003
- Experiments with neutralizing antibodies against IL 5  [ **IL5** ]  and GM CSF, revealed that IL 5  [ **IL5** ]  was the principal cytokine responsible for the down regulation of the IL 5R alpha  [ **IL5RA** ] .  Ref: 12519389 Clin Exp Immunol, 2003
- In conclusion our data indicate that IL 5  [ **IL5** ] , as opposed to its proposed action on eosinophil progenitors, down regulates the IL 5R alpha  [ **IL5RA** ]  chain on mature eosinophils.  Ref: 12519389 Clin Exp Immunol, 2003
- These aspects of IL 5  [ **IL5** ]  action on IL 5R alpha  [ **IL5RA** ]  expression could gain new insights into the mechanisms of specific immuno modulatory therapies, such as anti IL 5  [ **IL5** ] .  Ref: 12519389 Clin Exp Immunol, 2003

**Non-interactive relationship** (e.g. studied together, co-existance, homology, etc.)

- Incubation of eosinophils with IL 3, IL 5  [ **IL5** ] , or GM CSF led to reduced expression of IL 5R alpha  [ **IL5RA** ] , which was sustained for up to 5 days.  Ref: 12759409 J Immunol, 2003
- RT PCR was used to evaluate IL 5  [ **IL5** ]  receptor alpha IL 5R alpha  [ **IL5RA** ]  expression in various tissues and to assess bronchus and saphenous vein eosinophils through use of CCR3 expression.  Ref: 11897983 J Allergy Clin Immunol, 2002
- The aim was to study in vitro regulation of the IL 5  [ **IL5** ]  receptor alpha IL 5R alpha  [ **IL5RA** ]  on purified peripheral blood eosinophils from healthy subjects.  Ref: 12519389 Clin Exp Immunol, 2003
